# Global and regional left ventricular myocardial deformation measures by magnetic resonance feature tracking in healthy volunteers: comparison with tagging and relevance of gender

**DOI:** 10.1186/1532-429X-15-8

**Published:** 2013-01-18

**Authors:** Daniel Augustine, Adam J Lewandowski, Merzaka Lazdam, Aitzaz Rai, Jane Francis, Saul Myerson, Alison Noble, Harald Becher, Stefan Neubauer, Steffen E Petersen, Paul Leeson

**Affiliations:** 1 Oxford Cardiovascular Clinical Research Facility, Department of Cardiovascular Medicine, John Radcliffe Hospital, University of Oxford, Oxford OX3 9DU, UK; 2 Oxford Centre for Clinical Magnetic Resonance Research, Department of Cardiovascular Medicine, University of Oxford, Oxford, UK; 3 Institute of Biomedical Engineering, University of Oxford, Oxford, UK; 4 Mazankowski Alberta Heart Institute, University of Alberta, Edmonton, Canada; 5 William Harvey Research Institute, Barts and the London NIHR CVBRU, Queen Mary, University of London, London, UK

**Keywords:** Cardiovascular magnetic resonance, Feature tracking, Tagging, Strain, Myocardial displacement, Myocardial velocity

## Abstract

**Background:**

Feature Tracking software offers measurements of myocardial strain, velocities and displacement from cine cardiovascular magnetic resonance (CMR) images. We used it to record deformation parameters in healthy adults and compared values to those obtained by tagging.

**Methods:**

We used TomTec 2D Cardiac Performance Analysis software to derive global, regional and segmental myocardial deformation parameters in 145 healthy volunteers who had steady state free precession (SSFP) cine left ventricular short (basal, mid and apical levels) and long axis views (horizontal long axis, vertical long axis and left ventricular out flow tract) obtained on a 1.5 T Siemens Sonata scanner. 20 subjects also had tagged acquisitions and we compared global and regional deformation values obtained from these with those from Feature Tracking.

**Results:**

For globally averaged measurements of strain, only those measured circumferentially in short axis slices showed reasonably good levels of agreement between FT and tagging (limits of agreement −0.06 to 0.04). Longitudinal strain showed wide limits of agreement (−0.16 to 0.03) with evidence of overestimation of strain by FT relative to tagging as the mean of both measures increased. Radial strain was systematically overestimated by FT relative to tagging with very wide limits of agreement extending to as much as 100% of the mean value (−0.01 to 0.23). Reproducibility showed similar relative trends with acceptable global inter-observer variability for circumferential measures (coefficient of variation 4.9%) but poor reproducibility in the radial direction (coefficient of variation 32.3%). Ranges for deformation parameters varied between basal, mid and apical LV levels with higher levels at base compared to apex, and between genders by both FT and tagging.

**Conclusions:**

FT measurements of circumferential but not longitudinally or radially directed global strain showed reasonable agreement with tagging and acceptable inter-observer reproducibility. We record provisional ranges of FT deformation parameters at global, regional and segmental levels. They show evidence of variation with gender and myocardial region in the volunteers studied, but have yet to be compared with tagging measurements at the segmental level.

## Background

Left ventricular myocardial systolic strain and deformation parameters alter early in disease pathogenesis [1,2] and vary with cardiac pathologies [3,4]. These parameters can be measured with cardiovascular magnetic resonance (CMR) using a ‘tagging’ technique, in which magnetization saturation bands in a grid format are placed onto the myocardium at the start of the cardiac cycle. Image processing is then often performed using harmonic phase (HARP) imaging [5]. However, this can be difficult as tagged images have lower temporal resolution and the tag overlay fades through the cardiac cycle. A new software system, ‘Feature Tracking’ (2D Cardiac Performance Analysis, Tom Tec, Germany) aims to measure left ventricular deformation directly from SSFP cine CMR images, without the need for specialised tagged images. The software tracks features, such as the apparent cavity boundary or tissue patterns, related to the endocardial contour. The movement of features from frame-to-frame are used to quantify myocardial deformation over the cardiac cycle.

Feature Tracking has been used to quantify myocardial strain at a global level and within individual short axis slices in several studies [6-8]. However, clinical scenarios such as stress imaging or dyssynchrony evaluation need to measure strain regionally or even at a segmental level, and determine whether measured deformation parameters differ from normal values. We performed Feature Tracking analysis on cine CMR images obtained in a large number of normal subjects that gave outputs for deformation parameters including, strain, displacement, velocity and twist at a regional and segmental level. We evaluated reproducibility of selected outputs and assessed whether they showed variation according to myocardial region or between genders. Finally we compared values to those obtained by traditional tagging techniques.

## Methods

### Study population

The CMR images from one hundred and forty five healthy volunteers, recruited by advertisement as controls for research studies over a two year period, were analysed. None of the subjects had documented cardiovascular risk factors, cardiac disease or other medical problems relevant to cardiac function. All subjects had undergone the same non-contrast, left ventricular, SSFP CMR acquisition protocol on a Siemens 1.5 T Sonata scanner. Anthropometric measurements (height and weight), blood pressure and fasting blood tests (lipid profiles and glucose) had been obtained at the time of the CMR scan. The research studies were approved by the local ethics committee and informed consent for participation obtained from all subjects.

### Image acquisition

#### Cardiac magnetic resonance imaging

All images were recorded at 1.5 T with a 16 channel receiver coil without the use of contrast following the same standardisation protocol for all acquisitions. Image acquisition was prospectively electrocardiogram (ECG) gated with a precordial three lead ECG and acquired during end-expiratory breath holding. SSFP cine sequences were used to acquire localisation images followed by a SSFP ventricular short axis stack to obtain coverage of the entire left ventricle (LV) and horizontal long axis (HLA), vertical long axis (VLA) and left ventricular outflow tract views (LVOT) cine in 1 cm slices. Image acquisition parameters were echo time (TE) of 1.5 ms, a repetition time (TR) of 3.0 ms, temporal resolution 39.0 ± 2.8 ms and a flip angle of 60º, field of view 360 mm, slice thickness 8 mm, acquisition window 800 msec. In 20 subjects, a gradient echo-based tagging pulse sequence had also been performed in the long axis (horizontal long axis, vertical long axis and 3 chamber view) and in the basal, mid ventricular and apical short axis slices with a segmented K-space, multi-shot sequence (repetition time 25 ms, echo time 7.4 ms and flip angle 25º). Slice positions were chosen from the images obtained for the left ventricular short axis SSFP stack. The nearest slice to the base in which a complete circle of myocardium was visible throughout the cardiac cycle was selected as the basal slice. The mid-ventricular and apical slices were then selected with sequential 2 cm gaps towards to the apex [9].

### Image processing

#### Feature tracking analysis

2D Cardiac Performance Analysis Software (TomTec, Germany) was used to obtain strain quantification directly from cine images. The same experienced operator performed all measures following a standard protocol taught by the software manufacturer. In 12 subjects measures were repeated after an interval of 3 weeks by both this observer and a second experienced observer to assess inter and intra-observer agreement for measures. Following uploading of the image, the brightness is optimised to ensure optimal endocardial / blood pool discrimination. Points are placed along the endocardial border (for determination of longitudinal and circumferential deformation parameters) and both the epicardial and endocardial borders (for determination of the radial deformation parameters). The software then takes an endocardial border line through the marked points and searches for the most closely matching features along its length in subsequent frames. In a proportion of subjects it was visually apparent the software failed to track myocardial motion in certain segments. Poor tracking was considered to be present when the movement of the points along a portion of the border deviated from the movement of the apparent endocardial border by more than 50% of the myocardial width. A record was kept of which segments failed to track and these segments excluded from subsequent analysis. Longitudinal strain, strain rates, velocities and displacement were obtained from the long axis views. Circumferential and radial strain, strain rates, velocities as well as basal and apical rotation and rotation rates were measured from the short axis SSFP views. Short axis slice position was selected in the same way as for tagging image acquisition and, therefore, corresponding slices were used in those who had both sets of measures. Values were recorded at a segmental, regional level (basal, mid and apical) and global level.

#### Cardiovascular magnetic resonance tagging

In those subjects who had also undergone tagging studies semi-automated analysis of the tagged cine images was performed using CIM software (CIMTag2D v.7, Auckland MRI Research Group, New Zealand). A grid was aligned automatically to the myocardial tagging planes at end diastole. End systole is determined visually, and tags are adjusted at each frame through the cardiac cycle. Circumferential, longitudinal and radial myocardial strains and strain rates were calculated by the software from the motion of the intersected tag lines. Global values were recorded. Regional values were calculated at basal, mid and apical levels.

### Statistical analysis

Summary variables for subject characteristics and normal ranges of deformation parameters are presented as mean ± standard deviation (SD). Inter and intra observer variability was assessed using the coefficient of variation (CV). Comparison of demographic, clinical and myocardial deformation data between genders was performed by paired Student t test for normally distributed variables and Wilcoxonian test for non-normally distributed variables. Distribution of the variables was assessed using the Kolomogrov Smirnov test. Comparisons of myocardial deformation parameters between myocardial regions was performed by ANOVA with a repeated measure design using a Greenhouse-Geisser correction followed by paired Student t-test to define the differences. Comparison of feature tracking derived values with tagging results was assessed using Bland Altman [10] analysis. Initially the presence of proportional bias was assessed by performing linear regression. If the slope of the regression differed significantly from zero then the data was log transformed prior to performing the Bland Altman analysis to obtain the bias and limits of agreement which were then back transformed to give representative results.

## Results

### Study population and strain analysis

Baseline characteristics of the 145 subjects in the study cohort are recorded in Table 1. All subjects had analysable scans and of the 5200 segments assessed only 520 could not be tracked adequately by the software (10%). This was predominantly a problem with the segmental analysis of radially and longitudinally directed deformations, affecting 291 and 211 segments, respectively. Only 18 segments were considered unsuitable for analysis of circumferential strain. Inter and intra-observer agreements for Feature Tracking analysis are shown in Table 2. For global and regional strain measurements, the best observer agreement tended to be with circumferential strain at both global (CV 2.8-4.9%) and regional (CV range between 3.2% and 9.2%) levels with the poorest agreement for radial strain (global CV 22.9-32.3%; regional CVs range from 13.5 to 48.5%). Regional reproducibility was best at the mid and basal ventricular levels (with an inter-observer CV for mid circumferential strain of 4.5%) and worst at the apex.

**Table 1:** Baseline characteristics

**Characteristic**	**Overall**
Male (Total number)	54
Female (Total number)	91
Age (Years)	29.7 ± 7.6
Weight (kg)	70.7 ± 13.6
Height (cm)	171.2 ± 9.1
Body mass index	24.1 ± 4.4
Fasting total cholesterol (mmol/l)	4.5 ± 1.1
Fasting glucose (mmol/l)	4.8 ± 0.5
Systolic blood pressure (mmHg)	116.3 ± 11.9
Diastolic blood pressure (mmHg).	70.6 ± 8.2
Heart rate (beats/min)	66.9 ± 9.3

Data are presented as mean ± SD. KG, kilograms; CM, centimetres; mmol/l, millimoles/litre; mmHg, millimeters of mercury; min, minute.

**Table 2:** Feature Tracking, interobserver and intraobserver coefficient of variation

	**Global**		**Basal**		**Mid**		**Apical**	
	**FT Interobserver agreement**	**FT Intraobserver agreement**	**FT Interobserver agreement**	**FT Intraobserver agreement**	**FT Interobserver agreement**	**FT Intraobserver agreement**	**FT Interobserver agreement**	**FT Intraobserver agreement**
Longitudinal Strain	10.9	12.3	10.8	17.7	17.5	17.7	31.3	42.7
Longitudinal Strain Rate	16.2	16.0	34.3	19.2	21.1	17.8	25.6	23.2
Radial Strain	32.3	22.9	13.5	48.5	26.3	14.8	29.1	23.9
Radial Strain Rate	14.9	15.6	15.8	14.1	27.2	11.3	31.3	30.2
Circumferential Strain	4.9	2.8	3.2	6.0	4.5	6.4	9.2	6.0
Circumferential Strain rate	7.9	6.3	15.9	6.3	6.9	18.3	17.3	9.1
Longitudinal Velocity	13.2	22.2	24.3	23.3	33.7	32.2	65.5	31.2
Longitudinal Displacement	18.6	31.8	25.6	18.1	37.2	34.9	43.9	75.6
Radial Velocity	2.4	6.2	5.2	5.1	5.0	4.5	6.2	7.3
Radial Displacement	2.7	4.3	7.5	4.3	6.4	4.5	7.5	5.7

CMR indicates cardiac magnetic resonance; FT, feature tracking.

### Global and regional feature tracking strain deformation values

Ranges for deformation parameters derived by Feature Tracking at a global and regional level are shown in Table 3. The results at the segmental level are shown in Table 4. Interestingly, all longitudinal, circumferential and radial parameters were higher at the basal compared to the apical level (p < 0.05) although the magnitude of difference for circumferential strain did not meet significance (p = 0.09). Absolute rotation and rotation rate were also higher at the base compared with the apex (p < 0.05). Table 5 presents recorded values for global deformation parameters by gender with groups matched to ensure similar age distributions. There were no significant differences between genders in circumferential strain or strain rate. However, longitudinal strain values were higher in females, whereas, radial values were higher in males for strain (0.23 ± 0.04 vs. 0.22 ± 0.06, p = 0.02), strain rate (1.16 ± 0.17 s^-1^ vs. 1.13 ± 0.49 s^-1^, p = 0.03), velocities (2.60 ± 0.29 cm/s vs. 2.29 ± 0.28, p < 0.001) and displacement (5.24 ± 0.60 vs. 4.76 ± 0.69, p < 0.001).

**Table 3:** Values for systolic deformation parameters obtained using feature tracking for global and slice values in the volunteers studied (basal, mid, apical)

	**Longitudinal**				**Radial**				**Circumferential**		**Rotation (deg)**	**Rotation rate (deg/s)**	**Torsion (deg)**
**Level**	**Strain**	**Strain rate (s**^**-1**^**)**	**Velocity (cm/s)**	**Displacement (cm)**	**Strain**	**Strain rate (s**^**-1**^**)**	**Velocity (cm/s)**	**Displacement (cm)**	**Strain**	**Strain rate (s**^**-1**^**)**			
Global	−0.19 ± 0.03	−1.08 ± 0.24	2.60 ± 0.55	5.04 ± 1.14	0.25 ± 0.06	1.25 ± 0.4	2.5 ± 0.36	5.1 ± .073	−0.21 ± 0.03	1.21 ± 0.18	N/A	N/A	15.52 ± 7.55
Basal	−0.21 ± 0.05	−1.21 ± 0.36	3.38 ± 0.72	6.61 ± 1.83	0.26 ± 0.08	1.23 ± 0.39	2.84 ± 0.53	6.02 ± 1.08	−0.22 ± 0.04	−1.33 ± 0.28	−8.44 ± 6.06	−59.79 ± 33.44	N/A
Mid	−0.19 ± 0.04	−1.08 ± 0.27	2.65 ± 0.69	6.37 ± 10.15	0.24 ± 0.08	1.25 ± 0.36	2.48 ± 0.41	4.89 ± 0.82	−0.18 ± 0.03	−1.05 ± .018	N/A	N/A	N/A
Apical	−0.16 ± 0.05	−0.98 ± 0.34	1.7 ± 0.74	2.74 ± 1.15	0.23 ± 0.09	1.18 ± 0.43	2.19 ± 0.41	4.38 ± 0.82	−0.21 ± 0.38	−1.26 ±0.25	7.36 ± 5.38	52.90 ± 28.78	N/A

Data are presented as mean ± SD. s, seconds; cm, centimetres; deg, degrees; N/A, not applicable.

**Table 4:** Segmental values for systolic deformation parameters obtained using feature tracking in the volunteers studied

	**Longitudinal**				**Radial**				**Circumferential**	
**Level**	**Strain**	**Strain rate (s**^**-1**^**)**	**Vel (cm/s)**	**Displacement (cm)**	**Strain**	**Strain rate (s**^**-1**^**)**	**Vel (cm/s)**	**Displacement (cm)**	**Strain**	**Strain rate (s**^**-1**^**)**
Basal										
Anterior	−0.21 ± 0.10	−1.08 ± 0.88	4.13 ± 1.83	8.15 ± 4.54	0.39 ± 0.21	1.73 ± 0.81	3.12 ± 0.99	6.69 ± 2.08	−0.22 ± 0.08	−1.06 ± 2.77
Lateral	−0.20 ± 0.11	−1.61 ± 3.82	3.29 ± 1.55	5.85 ± 3.25	0.35 ± 0.17	1.93 ± 0.35	2.88 ± 0.75	5.89 ± 1.70	−0.26 ± 0.10	−1.59 ± 0.69
Posterior	−0.24 ± 0.11	−1.49 ± 0.69	3.72 ± 1.75	7.01 ± 3.00	0.26 ± 0.14	1.18 ± 0.56	3.01 ± 0.81	6.24 ± 1.69	−0.23 ± 0.08	−1.41 ± 0.58
Inferior	−0.16 ± 0.09	−0.87 ± 0.58	2.64 ± 1.15	5.16 ± 2.86	0.17 ± 0.11	0.93 ± 0.49	3.03 ± 0.9	6.51 ± 1.91	−0.20 ± 0.09	−1.21 ± 0.57
Septum	−0.15 ± 0.08	−1.08 ± 0.59	3.55 ± 1.73	5.86 ± 3.31	0.12 ± 0.08	0.81 ± 0.49	2.38 ± 0.75	5.04 ± 1.57	−0.22 ± 0.09	−1.26 ± 0.62
Anterior Septum	−0.24 ± 0.12	−1.5 ± 0.77	3.29 ± 1.81	7.30 ± 3.89	0.22 ± 0.12	1.13 ± 0.69	2.6 ± 0.8	5.67 ± 1.65	−0.20 ± 0.07	−1.20 ± 0.57
Mid										
Anterior	−0.23 ± 0.08	−1.21 ± 0.61	3.36 ± 1.72	6.05 ± 3.17	0.33 ± 0.18	1.61 ± 0.74	2.45 ± 0.58	4.92 ± 1.15	−0.18 ± 0.06	−1.05 ± 0.38
Lateral	−0.22 ± 0.11	−1.36 ± 0.74	2.98 ± 1.58	4.17 ± 2.9	0.32 ± 0.14	1.69 ± 0.8	2.6 ± 0.56	5.05 ± 1.12	−0.19 ± 0.06	−1.09 ± 0.45
Posterior	−0.18 ± 0.09	−1.14 ± 0.67	2.59 ± 1.33	4.68 ± 2.58	0.26 ± 0.13	1.25 ± 0.5	2.56 ± 0.65	4.95 ± 1.3	−0.18 ± 0.06	−1.10 ± 0.37
Inferior	−0.13 ± 0.07	−0.73 ± 0.42	2.64 ± 1.15	4.86 ± 2.58	0.16 ± 0.09	0.89 ± 0.44	2.50 ± 0.60	4.9 ± 1.22	−0.17 ± 0.06	−1.05 ± 0.37
Septum	−0.15 ± 0.08	−0.91 ± 0.51	2.97 ± 1.57	4.14 ± 2.26	0.13 ± 0.08	0.85 ± 0.46	2.38 ± 0.6	4.67 ± 1.22	−0.18 ± 0.06	−1.04 ± 0.37
Anterior Septum	−0.19 ± 0.12	−1.22 ± 0.83	2.49 ± 1.37	4.79 ± 2.1	0.22 ± 0.13	1.25 ± 0.64	2.41 ± 0.59	4.83 ± 1.14	−0.17 ± 0.06	−0.93 ± 0.38
Apical										
Anterior	−0.18 ± 0.07	−1.11 ± 0.60	1.49 ± 0.77	2.66 ± 1.39	0.29 ± 0.14	1.42 ± 0.61	2.09 ± 0.51	4.20 ± 0.98	−0.20 ± 0.05	−1.12 ± 0.34
Lateral	−0.13 ± 0.07	−0.83 ± 0.43	3.07 ± 2.33	2.37 ± 2.03	0.24 ± 0.14	1.28 ± 0.60	2.27 ± 0.54	4.48 ± 1.06	−0.22 ± 0.05	−1.31 ± 0.43
Inferior	−0.16 ± 0.07	−0.9 ± 0.44	1.53 ± 0.88	3.19 ± 1.81	0.14 ± 0.12	0.90 ± 0.55	2.30 ± 0.53	4.60 ± 0.91	−0.23 ± 0.07	−1.38 ± 0.47
Septum	−0.13 ± 0.07	−0.88 ± 0.46	1.95 ± 1.24	2.33 ± 1.43	0.18 ± 0.10	1.07 ± 0.57	2.09 ± 0.51	4.22 ± 1.05	−0.21 ± 0.07	−1.21 ± 0.48

Data are presented as mean ± SD. s, seconds; cm, centimetres; vel, velocity.

**Table 5:** Deformation results from feature tracking and tagging according to gender

	**CMR FT**			**CMR Tagging**		
	**Male (n = 54)**	**Female (n = 62)**	**P Value**	**Male (n = 7)**	**Female (n = 13)**	**P Value**
Age	27.46 ± 5.06	26.59 ± 4.64	0.29	26.76 ± 1.53	27.57 ± 1.51	0.35
Systolic BP	115.61 ± 10.61	112.96 ± 10.74	0.24	110.31 ± 5.87	116.42 ± 12.67	0.35
Diastolic BP	68.15 ± 7.45	69.98 ± 6.99	0.25	66.61 ± 6.65	69.42 ± 4.31	0.35
BMI	23.99 ± 3.06	23.11 ± 4.03	0.02	23.89 ± 3.20	21.26 ± 2.11	0.06
LV Mass Index	63.83 ± 5.07	49.27 ± 7.93	<0.001	57.45 ± 9.16	42.34 ± 5.99	0.002
EF CMR	63.88 ± 5.07	64.35 ± 5.23	0.62	63.76 ± 4.56	65.42 ± 4.57	0.28
Longitudinal Strain	−0.17 ± 0.04	−0.18 ± 0.04	0.04	−0.14 ± 0.01	−0.16 ± 0.01	0.005
Longitudinal Strain Rate (s^-1^)	−0.98 ± 0.28	−1.13 ± 0.31	0.02	−0.69 ± 0.66	−0.81 ± 0.12	0.09
Circumferential Strain	−0.20 ± 0.02	−0.21 ± 0.03	0.86	−0.19 ± 0.02	−0.19 ± 0.02	0.08
Circumferential Strain Rate (s^-1^)	−1.19 ± 0.16	−1.16 ± 0.15	0.31	−0.84 ± −0.10	−0.91 ± 0.64	0.12
Radial Strain	0.23 ± 0.04	0.22 ± 0.06	0.02	0.15 ± 0.04	0.10 ± 0.02	0.003
Radial Strain Rate (s^-1^)	1.16 ± 0.17	1.13 ± 0.49	0.03	0.98 ± 0.31	0.65 ± 0.89	0.014
Radial Velocity (cm/s)	2.60 ± 0.29	2.29 ± 0.28	<0.001			
Radial Displacement (mm)	5.24 ± 0.60	4.76 ± 0.69	<0.001			
Longitudinal Velocity (cm/s)	3.04 ± 0.91	3.14 ± 1.06	0.64			
Longitudinal Displacement (mm)	4.51 ± 1.91	4.90 ± 1.98	0.28			

Data are presented as mean ± SD. s, seconds; cm, centimetres; mm, millimetres; BP, blood pressure; LV, left ventricle; EF, ejection fraction; BMI, body mass index.

### Feature tracking and tagging comparison

**Figure 1:**
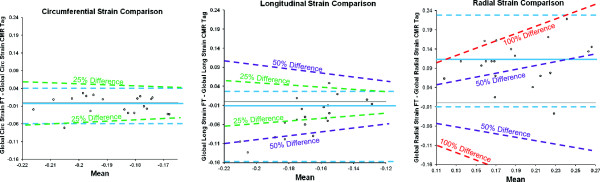
**Modified Bland Altman plots showing comparisons between Feature Tracking and CMR tagging for global strain parameters: Circumferential strain (left); longitudinal strain (middle); radial strain (right).** The bias (blue solid line) and limits of agreement (blue dashed lines) are shown. The oblique dashed lines demonstrate 25 (green), 50 (purple) and 100% (red) difference levels.

Analysis of a complete data set using Feature Tracking was quicker than by tagging (8.8 ± 4.7 minutes vs. 15.4 ± 4.9 minutes, p < 0.05). The plots of Figure 1 show evidence of differences between the Feature Tracking and tagging methods of strain measurement, particularly for measurements of longitudinal and radial strain. Comparing the longitudinal measures of strain, about half of the points showed >25% differences of global values, greater deformation being associated with greater overestimation of the negative strain by FT relative to tagging. Comparing radial measures of strain, more than half of the points showed between 50 and 100% differences, with all points except one showing overestimation of strain by FT relative to tagging, and a trend suggestive of greater overestimation when there is greater strain.

Absolute values for tagging derived strain for each gender are presented in Table 5. The different patterns in regional strain between genders was similar to those described for Feature Tracking. However, absolute values of reported strain differed, in particular for radial strain in females. This is demonstrated in the Bland Altman analysis which is presented in Table 6 for both global and regional parameters. For global strain parameters the narrowest limits of agreement were seen for circumferential strain (−0.06 to 0.04) with a systematic bias in radial strain and wide limits of agreement (−0.01 to 0.23). A similar pattern was seen when comparing strain rates estimated by feature tracking and those by tagging with the lowest bias and narrowest limits of agreement being seen with circumferential strain rate (−0.21 s^-1^, -0.53 to 0.11) and the poorest agreement with radial strain rate (0.26 s^-1^, -0.34 to 0.86). Larger biases and limits of agreement were seen when comparing feature tracking with tagging at a regional level compared with a global level although again, the agreement for radial strain was poorest.

**Table 6:** Bland Altman analysis for comparison between CMR tagging and feature tracking

	**CMR Tagging vs. FT agreement**							
	**Global**		**Basal**		**Mid**		**Apical**	
Variable	Bias	LOA	Bias	LOA	Bias	LOA	Bias	LOA
Longitudinal Strain	−0.01	−0.16 to 0.03	−0.06	−0.19 to 0.06	−0.05	−0.21 to 0.11	0.04	−0.12 to 0.20
Longitudinal Strain Rate (s^-1^)	−0.22	−0.82 to 0.37	0.01	−0.16 to 0.19	0.03	−0.05 to 0.12	−0.02	−0.12 to 0.07
Radial Strain	0.11	−0.01 to 0.23	0.12	0.03 to 0.23	0.12	−0.05 to 0.30	0.08	−0.13 to 0.30
Radial Strain Rate (s^-1^)	0.26	−0.34 to 0.86	0.20	−0.71 to 1.11	0.41	−0.32 to 1.16	0.17	−0.83 to 1.16
Circumferential Strain	−0.007	−0.06 to 0.04	−0.05	−0.14 to 0.04	0.02	−0.04 to 0.07	0.009	−0.05 to 0.07
Circumferential Strain rate (s^-1^)	−0.21	−0.53 to 0.11	−0.44	−1.09 to 0.21	−0.07	−0.42 to 0.27	−0.12	−0.50 to 0.25

CMR indicates cardiac magnetic resonance; FT, feature tracking; LOA, limits of agreement; s, seconds.

## Discussion

This study documents ranges for all left ventricular myocardial systolic strain parameters as well as myocardial displacement and velocities recorded using Feature Tracking down to a segmental level in a large group of healthy subjects, although the latter have yet to be compared with an alternative type of measurement. The results showed regional variation, with higher strain at the base than apex, as well as gender differences.

Feature Tracking software delivers outputs of myocardial strain, segmental velocity and displacement parameters which are relatively quick in terms of image acquisition and post processing. The technique avoids the additional time needed for either tissue phase mapping or tagging and raises the possibility of retrospective analysis of existing CMR datasets. We found the software could be easily applied to existing SSFP cine sequences and was apparently able to track 90% of imaged segments. This compares favourably to echocardiographic studies of regional speckle tracking analysis in which strain typically can only be measured in around 80% of segments [11-13]; presumably due to difficulties in obtaining adequate echocardiographic windows with poorer delineation of the endocardial and epicardial borders compared with CMR.

Nevertheless, as others have reported [7,14,15], we found observer reproducibility to vary considerably between the three main deformation directions with global strain values acceptable for circumferential assessment (inter-observer CV 4.9%) but poor for radial strain (CV 32.3%). There was also a deterioration in reproducibility from a global to regional level with poor reproducibility for apical measures. This pattern is similar to that previously reported for reproducibility using the tagging technique in which CVs for circumferential strain range from 8.3% to 10.8% and for radial strain from 9.0% to 59.2% [16]. It has been proposed that the poor reproducibility for radial parameters may be due to the geometry of the heart with analysis in a plane of movement with the smallest potential diameter for tracking. Our results suggest both tagging and feature tracking are similarly limited in the radial direction. This poor reproducibility may explain why we found significant variation in absolute deformation values recorded in the radial direction with tagging and feature tracking. This was particularly evident in females for whom mean radial strain by feature tracking was 0.22 ± 0.06 compared to 0.10 ± 0.02 by tagging. Alternatively, as there was a systematic bias, with larger values derived from feature tracking, this difference may be a real effect and relate to how strain is measured by the two techniques.

Tagging measures myocardial strain from the changing in-plane separations of tags that mark the intersections of orthogonally orientated tagging planes. They are therefore relatively unaffected by a through-plane component of motion. In contrast, feature tracking analyses motion in a 2D plane of features along a myocardial band defined by the endocardial border. The algorithms used by TomTec to track features are based on an adaptation of particle velocimetry algorithms in common use in multiple technologies including speckle tracking. They use voxel patterns within the image identified during initial contour application and subsequent searching between frames based on a hierarchical protocol that allows for reducing region of interest to improve accuracy, recognition of variation in motion between base and apex and rules regarding endocardial and epicardial boundaries [17]. Interestingly, the variation between feature tracking and tagging measures of longitudinal strain appears to vary with magnitude of strain so that the difference in measures is greater with larger strain values.

Changes in the voxel pattern during the cardiac cycle within the myocardium may have an impact on consistency of feature tracking and account for some variation in strain measures, particularly in the radial direction. For instance, it is possible the compaction and exclusion of blood from interstices in trabeculated myocardium at end systole may alter voxel appearances in this region sufficiently to make accurate tracking difficult. The higher degree of trabeculations seen at the LV apical level when compared with the basal level may account, in part, for the increased variability in measurements seen in this region.

These differences in the approach of feature tracking prompted our study to define feature tracking-specific ranges for strain [6-8,18]. Normal ranges of strain and velocities have already been described using various other imaging modalities including tagging [11,13,19-21] and significant variations noted. In the future, development of standardised reference ranges may allow convergence of technologies and ranges. However, significant further work is needed, including with Feature Tracking. For example, it is not known what effect contrast agents have on strain results and future validation of segmental strain values is necessary.

Our results showed some consistent trends between techniques in our study population. For instance, deformation values varied between genders and myocardial regions when assessed by both feature tracking and tagging [22,23]. Multi-modality imaging studies describing normal strain values have tended to vary in their findings with respect to differences between the base and apex [24] with some reports of lower strain values towards the apex [25]. However, the velocity values we obtained in this study are similar to previous reports both in terms of normal ranges and the finding of a reduction of myocardial velocities at the apex compared to the left ventricular base [26]. Circumferential measures, particularly at a global or mid-ventricular level showed the least inter-observer variability and the greatest comparability between techniques in our study. Furthermore, at the mid-ventricular level the circumferential strain values reported in our study are similar to those previously reported for feature tracking by Hor et al. (0.18 ± 0.03 vs. 0.19 ± 0.02) [6] and Harrild et al. (0.18 ± 0.03 vs. 0.21 ± 0.04) [9]). It has been suggested that a relatively simple measures such as relative change of boundary length may be equally robust to characterise circumferential myocardial deformation [14]).

## Conclusions

FT measurements of circumferential but not longitudinally or radially directed global strain showed reasonable agreement with tagging and acceptable inter-observer reproducibility. We recorded provisional ranges of FT deformation parameters at global, regional and segmental levels in healthy volunteers. The software rapidly extracts these measures from existing SSFP cine images. Our results show evidence of variation with gender and myocardial region in the volunteers studied, but have yet to be compared with tagging measurements at the segmental level.

## Abbreviations

CMR: Cardiac magnetic resonance; ECG: Electrocardiogram; HARP: Harmonic phase; HLA: Horizontal long axis; LOA: Limits of agreement; LV: Left ventricle; LVOT: Left ventricular outflow tract; SD: Standard deviation; SSFP: Steady state free precession; TE: Echo time; TR: Repetition time; VLA: Vertical long axis.

## Competing interests

The authors declare that they have no competing interests.

## Authors’ contributions

DA was involved in the conception and design of the study; acquisition of data, analysis, drafting and approved the manuscript. AL carried out acquisition of data, analysis, revising the draft critically for important intellectual content and has given final approval of the version to be published. ML was involved in acquisition of data, analysis and has given final approval of the version to be published. AR was involved in analysis and has given final approval of the version to be published. JF carried out acquisition of data, revising the draft critically for important intellectual content and has given final approval of the version to be published. SM was involved in revising the draft critically for important intellectual content and has given final approval of the version to be published. AN was involved in revising the draft critically for important intellectual content and has given final approval of the version to be published.SN was involved in revising the draft critically for important intellectual content and has given final approval of the version to be published. HB was involved in revising the draft critically for important intellectual content and has given final approval of the version to be published. SEP was involved in revising the draft critically for important intellectual content and has given final approval of the version to be published. PL was involved in the conception and design of the study; revising the draft critically for important intellectual content and has given final approval of the version to be published. All authors read and approved the final manuscript.

## Authors’ information

This work was funded by a grant from the Engineering and Physical Sciences Research Council (EP/G030693/1) and supported by the Oxford British Heart Foundation Centre of Research Excellence and the National Institute for Health Research Oxford Biomedical Research Centre. Dr P Leeson is supported by the British Heart Foundation. Dr S Petersen was directly funded by the National Institute for Health Research Barts and The London Biomedical Research Unit.
